# AI-assisted adaptive-optical robotic metrology for sub-millimeter localization and dimensional measurement of blind holes

**DOI:** 10.3389/frai.2026.1983856

**Published:** 2026-10-26

**Authors:** Giriraj Mannayee

**Affiliations:** Department of Design and Automation, School of Mechanical Engineering, Vellore Institute of Technology, Vellore, India

**Keywords:** adaptive illumination, artificial intelligence, blind-hole measurement, dimensional inspection, machine vision, optical sensing, precision manufacturing, robot-camera calibration

## Abstract

**Introduction:**

The process of measuring the dimensions of a blind hole is difficult because the interior surfaces and base are hard to probe using normal probes and camera equipment. Optical measurement is additionally affected by restricted visibility, lighting differences, reflectance, view angle, and robotic positioning accuracy.

**Methods:**

An adaptive robotic optical metrology system using AI is suggested to achieve non-contact hole inspection in this paper. The system consists of a six DOF robot, an optical measurement head with a camera, adaptive lighting system, AI segmentation, sub-pixel circle and ellipse detection, and robot-camera calibration with error correction using CMM reference data. The system was tested using 30 samples having holes with diameter ranges of 5-15 mm, depth range of 10-45 mm, aspect ratio of 2.0-3.1, three different surface reflectivity conditions, and angle range of 0-20°.

**Results:**

All trials were successfully carried out for detecting the holes. Average position error in the three-dimensional space was 0.0322 mm; the RMSE of position error was 0.0344 mm; the maximum position error was 0.0645 mm. The average absolute diameter error was 0.0544 mm; RMSE of the absolute diameter error was 0.0647 mm. Average repeatability was 0.0217 mm; average confidence in AI-based segmentation was 0.935. Average position errors varied between 0.026 mm and 0.037 mm depending on reflectivity classes and increased at 15° and higher approach angles.

**Discussion:**

The results offer initial proof-of-concept validation for the use of AI-aided adaptive optical robotic systems to perform automated and contactless inspections of blind holes at least within the current scope of operation. Nevertheless, the small number of samples used, single replication and partially confounding experimental conditions pose a limit on the generalizability of the results. Additional research is necessary using multiple trials, ablation tests of modules, and larger depth-to-diameter ratios are required to establish the system’s robustness and measurement reliability.

## 1 Introduction

The inspection of hole dimensions plays a critical role in precision machining, since the location, diameter, direction, depth, and form of holes determine the accuracy of assembling, the strength of parts, and their sealing capabilities. Many functional holes in aerospace parts, automotive parts, tools, hydraulic systems, medical devices, and additive manufacturing parts are blind holes. The examination of such holes becomes very complex as compared to through holes due to the obscured surfaces in the inner hole end. In addition, tactile and optical measurements along with the illumination become impossible due to depth, surface condition, and viewing angle. The coordinate measuring machine (CMM) is considered as the standard instrument of metrology used for dimensional inspection, and it is highly accurate. However, the conventional approach of CMM inspection requires the set up time and therefore cannot be used for rapid inspection. Contact measurement is also hindered by the form of the stylus, bending of the probe, and the probability of damaging surfaces on fragile components. There are already established standards like the ISO 10360 standard series that define the process of accepting and re-verifying contact, imaging, articulated arm, and optical coordinate measuring machines, which are the basis in this study. A non-contact machine vision method would be an ideal choice because it does not handle the work-piece physically. According to the most recent research done on robot vision, there is an incredible level of accuracy that is possible when there is optical access to the hole boundary: “Positioning accuracy of robotic binocular vision for recognizing the holes in satellite assembly was reported to be 0.06 mm.” In addition, the circular hole measurement method with radial pixel scanning, sub-pixel resolution, super-resolution technique and perspective error correction that was intended to be implemented by 2026 had 100% hole recognition with 0.016 mm mean error and 0.032 mm maximum error. The above data clearly indicates that optical metrology is quite mature for measuring common circular holes. But the issue of blind hole measurement is completely different. The inner surface of the hole does not have sufficient illumination. In addition, if the sensor is not aligned along the axis of the hole, then there will be a distortion in the edge location due to perspective. Further, there is the appearance of reflection effects because of the metallic or machined surface of the hole. Moreover, there is a degradation of the optical signal due to the increased depth of the hole relative to the diameter of the hole. The optical systems that have been designed for the purpose of detecting holes with small diameters are the endoscope, reflection imaging, and robust pixel calibration. However, it must be stated that this is a fundamentally different problem to that of external circular holes. One other aspect contributing to the uncertainty in this case is when the optical sensor is attached to an industrial robot rather than to a metrology frame. While the main advantages of using the industrial robot include the capability to manipulate the work environment and perhaps even the possibility of using the same machine to perform machining or assembly operations, the precision of the absolute positioning on the part of such a robot will be only one to two orders of magnitude less precise than that of the CMM. Calibration and compensation approaches have been the focus of previous studies on non-contact sensor technology attached to robots before using it in robotic hole measurements. Besides the metrology problem raised above, there is also the question of application of blind hole metrology in various industrial processes. In aerospace turbines and components, small and improperly positioned holes for fasteners and cooling channels decrease the fatigue resistance and assembly accuracy; in automotive engine and transmission housing castings, holes that have been drilled in machine lines should be checked without contact rapidly, and the time spent by conventional CMM is too long to provide inline control; and in machining and post processing stages in additive manufacturing, blind holes are typically the last measured feature of a part. Robot inspection based on the use of vision sensors looks promising for all three applications, as it can be readily mounted onto a machine or line, but, as shown by the two lines of research above, there is no method yet which would incorporate lighting as an active control parameter along with robot positioning inaccuracies compensation. Thus, the originality of this work is in the following three aspects: (i) the illumination level is adaptively tuned using a quantitative measure of the image quality, and not predefined, so that the illumination level depends on the depth and reflectivity of the hole; (ii) a trained AI-based segmentation algorithm, which uses a multi-objective loss function (segmentation, edge, and geometric losses), is employed for removing reflections from the inner walls of the blind hole, not the visible boundary of the hole, as was done in the past optical studies; and (iii) the combination of the above described optical signal processing sequence with a data-driven robot error correction model, which means that the accuracy experiment results are not an optical setup anymore but a robot-based sensing system. Sections 3 through 5 show how this combination takes place, while section 6 highlights the limitations of this approach and the need for a hardware-based validation study.

These dual challenges are addressed within this paper via an AI-based metrological architecture which uses both optics and robotics. This method not only depends upon the naturally occurring visibility of the external hole boundary but also introduces controlled illumination to the invisible hole and considers its optical reaction as a measurable quantity. The adaptive illumination control determines the emission based on the depth of holes and reflectance of the surface; the AI-powered segmentation network then segregates valid optic data from reflection artifacts and noise; the boundary is extracted at sub-pixel precision, and fitted into a circle or ellipse; and finally the coordinates of the center are mapped into the robot base coordinate system using the camera-robot calibration and error compensation based on the learning model using the ground truth obtained from CMM.

### 1.1 Contributions

This paper makes six key contributions. The first contribution consists of formulating an adaptive light emission criterion that produces a stable and depth/reflectivity compensated optical behavior in a blind hole using an illumination quality objective function that is optimized in closed loop. Secondly, a technique of optical feature extraction using AI-based technology, which includes segmentation-edge-geometry loss function, is used to detect blind holes under varying illumination and reflectance conditions. Thirdly, sub-pixel circle/ellipse geometry reconstruction along with camera calibration is employed to estimate hole center, radius, and orientation at oblique angles. Fourth, an AI-robot coordinate transformation and error compensation framework based on the AI measurements with respect to CMM measurements is created. Fifth, a multifactor experiment design incorporating hole diameter, depth, surface reflection, and robot approach angle with a 30-experiment test that includes position error, diameter error, repeatability, detection rate, and AI confidence is designed. Sixthly, Preliminary quantitative results are provided to show that the accuracy of measurement was constant across reflectivity groups due to adaptive illumination, while correlation analysis has shown that the depth of the holes is the key variable behind the decreasing confidence level of the AI. Central to the research is the hypothesis that adaptive optical excitation together with feature extraction using AI and geometric compensation for robots will enable robust non-contact sub-millimeter localization of blind hole geometry despite differences in depth, reflectivity, and angle of sensor operation. Thirty trials validation is a concept-proving study on a small sample. The rest of the paper is structured as follows. Section 2 reviews related work and positions the research gap. Section 3 gives the system architecture proposed along with the methodology involved in it, which is formulated through a set of equations. Section 4 discusses the experiment setup and the error measures. Section 5 gives the results of testing along with the discussion related to comparative analysis, correlation analysis, statistical-robustness analysis, and uncertainty analysis. Section 6 concludes the paper and outlines future work.

## 2 Background and related work

The existing literature concerning robotic blind-hole measurement may be divided into four categories, which include tactile metrology, traditional machine vision, highly accurate robotic vision, and blind hole optical measurement. Each individual group solves only part of the problem of measurement; however, when taken alone, none can solve the whole problem of blind geometry, illumination, reflectivity, and location of the robot.

### 2.1 Contact-based metrology

The metrology reference for dimensional measurements is CMMs and tactile probes and is used as the reference in this research, which is similar to the acceptance and verification procedures of ISO 10360 for contact, imaging, articulated arm, and optical coordinate measuring machines (Catalucci et al., 2022). As previously shown in a three-dimensional probing study, very small blind holes could be probed according to the mechanical stylus principle, although such measurement techniques rely on physical accessibility and compensation for the deflection of the probe tip, and are unsuitable for rapid in-process inspection of small or deep cavities.

### 2.2 Conventional machine vision

A vision system that employs fixed illumination along with global thresholding, Canny edge detection, and either circle or ellipse fitting can quickly detect hole center and size when the boundary is distinctly identifiable and the lighting conditions are fixed. Nonetheless, traditional thresholding is very dependent on illumination, surface texture, reflection, and contrast (Abosinnee et al., 2025; Yang et al., 2026). In addition, conventional thresholding rarely works for internal occlusion cavities without any modifications.

### 2.3 High-precision robotic vision

Modern research on robotic vision has shown sub-0.1 mm and, in a study from 2026, sub-0.02 mm error in the mean hole diameter estimation in conventional circular holes measurement with binocular vision, radial pixel scan, and super-resolution (Yang et al., 2026). The findings provide the limit of possible accuracy for the boundaries of holes that can be observed through light, yet largely ignore holes where there are difficulties associated with blind hole depth, illumination, and reflection from walls.

### 2.4 Deep-hole and blind-hole optical inspection

Optical instruments that are particularly designed for deep hole inspection with small diameters include endoscopic probing instruments, reflecting imaging systems, and optical or laser-based deep hole measuring tools, with particular focus on optical resolution, bright light robustness, and equivalence calibration of pixels. The use of robotic systems equipped with non-contact sensors to measure internal and/or hard-to-reach features has also been shown, and hence, robotic optical metrology can be feasible when robot calibration and compensation are considered explicitly. One set of literature which is very relevant is the literature on robotic positional accuracy in handling holes using force guided as well as tactile guided assembly of peg-in-hole. Literature exists on tactile guided exploration as well as measurement of contact wrench for localization of peg-in-hole target under uncertainty (Huang et al., 2026; Liao et al., 2025) as well as force based deep Q-learning (Markert et al., 2023; Liu et al., 2022). Force-tactile and visual-tactile combination methods make use of both touch and vision for pose estimation and insertion control (Xie et al., 2024; Negi et al., 2025; Wang et al., 2026), and active force-torque approaches have shown sub-millimeter localization of unseen holes (Wang et al., 2024). Relevant research includes hole search approaches in case of uncertain initial placement (Zang et al., 2025), multi-sensor snap fit assembly (Shen et al., 2025), time series force controlled assembly into inclined and flexible support holes (Mei et al., 2024; Wu et al., 2025), wrist force sensing based assembly in case of tight clearance (Jia et al., 2025), impedance controlled assembly by demonstrations (Xiong et al., 2026), and single demonstration assembly programming (Rugbani, 2026). In total, the literature provides proof that robotic systems have the ability to attain sub-millimeter positional accuracy when engaging holes through force/tactile sensing mechanisms; yet, all the above methodologies tackle contact-based peg insertion and not non-contact optical evaluation of the inner dimensions of blind holes. The current study is thus complementary to the above literature as it addresses the issue of the upstream problem, which involves determination of the location and dimensions of the blind hole center, diameter, and depth via optical means without carrying out any insertion process using the framework presented in section 3.

### 2.5 Research gap and novelty

These limitations have been summarized in Table 1 along with the particular component of the framework which is designed to overcome each such limitation.

**Table 1:** Research gaps and proposed framework elements.

Existing limitation	Proposed solution element
External hole boundary often unavailable	Internal light-emission-based optical feature generation
Illumination effectiveness varies with hole depth	Adaptive light-intensity control (section 3.5)
Specular reflection produces false edges	AI-based valid-region segmentation (section 3.6)
Pixel-level localization limits accuracy	Sub-pixel geometric estimation (section 3.7)
Robot positioning introduces global error	Robot-camera calibration and pose compensation (section 3.8–3.9)
Perspective changes apparent diameter	Calibrated ellipse-to-circle geometric reconstruction
Different materials produce different brightness	Reflectivity-aware adaptive illumination
Fixed thresholds are unreliable across conditions	Adaptive, confidence-based AI thresholding
Blind geometry restricts sensor access	Compact robot-mounted optical probe head
Conventional methods provide limited autonomy	Closed-loop robotic measurement with CMM-referenced compensation

Therefore, the gap that has been found can be defined as the lack of a common approach to integration of blind geometry, adaptive lighting, artificial intelligence segmentation, sub-pixel localization and robot calibration into a single measurement loop. The novel nature of this research can thus be described as follows: A closed-loop AI-driven robotic metrology system in which controllable light emission creates optical data within the blind hole, the AI model finds reliable optical data within the blind hole despite varying reflectivity, and robot-informed geometry reconstruction converts this optical data into sub-millimeter dimensional data that is ground- truth with CMMs. Since it has been found that existing research already shows an error of 0.016 mm in mean diameter of optically accessible circular holes, the scientific significance of this research does not lie in having higher accuracy in conventional holes, but in presenting a methodology that works effectively in the case of blind holes measurement under variable depth, reflectivity, and robot-induced uncertainty.

## 3 Proposed methodology

The process of measurement is divided into several stages: robot positioning, optical probing, adaptive illumination, imaging, segmentation by means of artificial intelligence algorithms, sub-pixel estimation of geometrical parameters, robot-camera calibration, errors elimination, and blind-hole measurement. The robot places the optical probe close to the object. Adaptive illumination allows taking a high-quality picture of the hole to avoid reflection and non-uniform illumination. AI-based segmentation technology is applied to process the captured image and detect the accurate boundary of the hole. The next step involves sub-pixel image processing to measure the hole center and size with more accuracy than the image resolution. Coordinates obtained through measurement are transformed from the camera coordinate system to the robot coordinate system through robot-camera calibration. Errors introduced by distortion of the camera, robot positioning, lighting, and calibration are subsequently corrected. The final step is to compute the coordinates and size of the blind hole without making any contact. The method is discussed employing metrology and computer vision formulae, which are numbered sequentially to facilitate their referencing in the discussion section.

### 3.1 System architecture

The measurement setup is structured in seven layers of functionality and is shown in Figure 1. The first layer is a six-axis industrial robotic manipulator used to place the optical measurement head in relation to the object being measured, offering its pose ᴮTe relative to the robot base coordinate frame {B}. The second layer is the optical measurement head, which comprises a small industrial camera, a low distortion (telecentric) lens, a source of LED or laser light, an optical diffuser, an optical window, and optionally, a depth position measurement device; it is built for approaching the blind hole opening without touching its inside surface. Layer 3 is the light-emission adaptive layer that controls the intensity of light emission based on the depth of the hole and the surface reflectivity. The fourth layer is the AI vision layer that processes the acquired image into a probability map of the valid optical region. The fifth layer conducts the sub-pixel geometric reconstruction of the hole’s center, diameter, and axis. Layer 6 deals with robot aware coordinate transformation and systematic error compensation using CMM ground truth.

**Figure 1:**
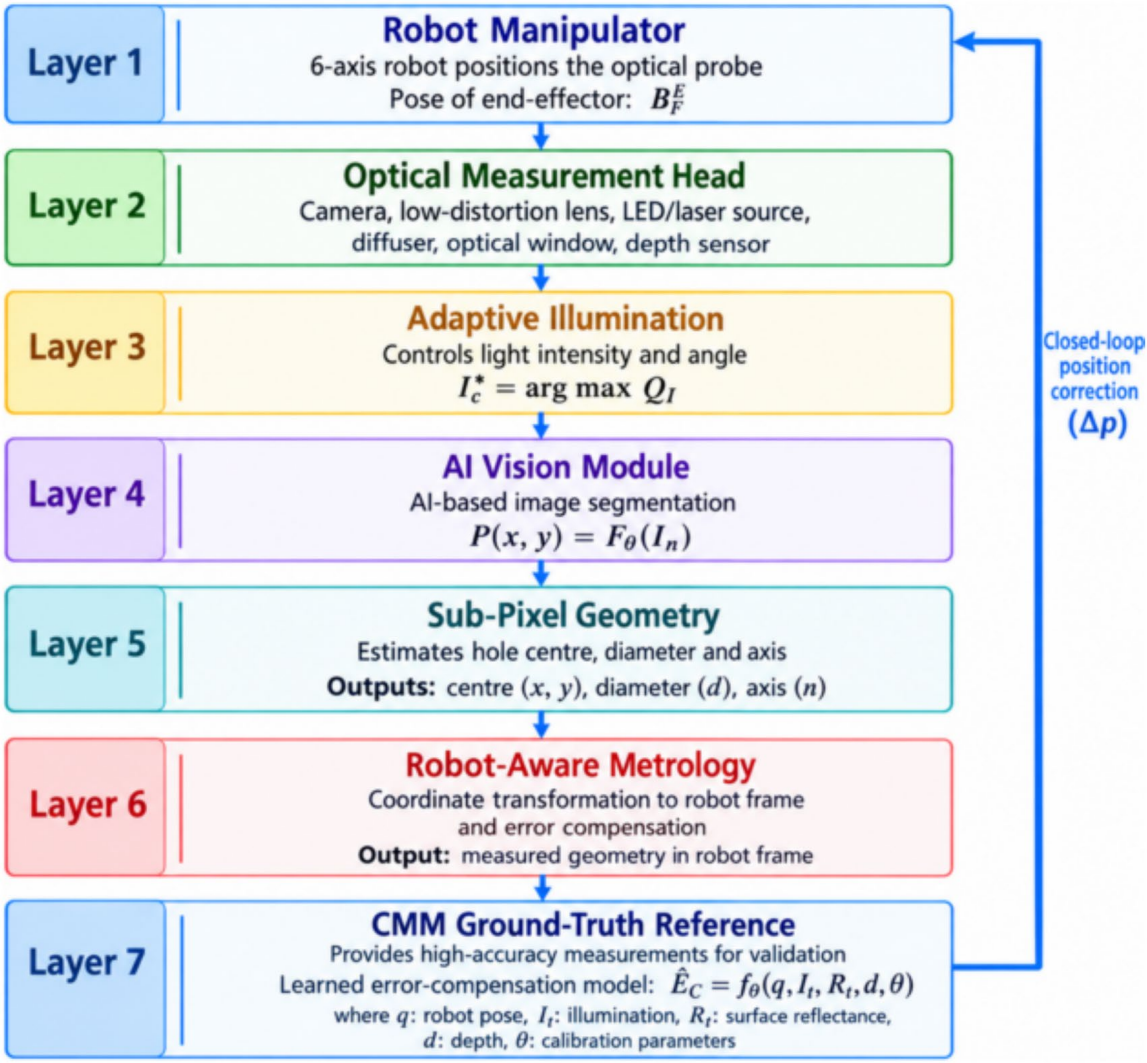
AI-assisted robotic blind-hole measurement system architecture.

### 3.2 Optical measurement principle

Physical principle:

Intrinsic property of the material (the hole interior): optical response when irradiated with a well-defined source. Simplified diffuse reflection: The reflected intensity I*_r_* is a function of the emitted intensity I_e_, surface reflectance R_s_ and angle of incidence θ through


$$I_{r}\propto I_{e}\;R_{s}\;\cos\theta$$ (1)

Actual machined or metal surfaces possess both diffuse and specular reflection elements, and therefore the real value of the image intensity I_m_ can be more correctly expressed as a function of intensity, reflectance, optical depth d, surface state ρ, illumination wavelength λ, and noise ε:


$$I_{m}=f(I_{e},R_{s},d,\theta ,\rho ,\lambda )+\varepsilon$$ (2)

Figure 2 represents this light arrangement within the blind hole.

**Figure 2:**
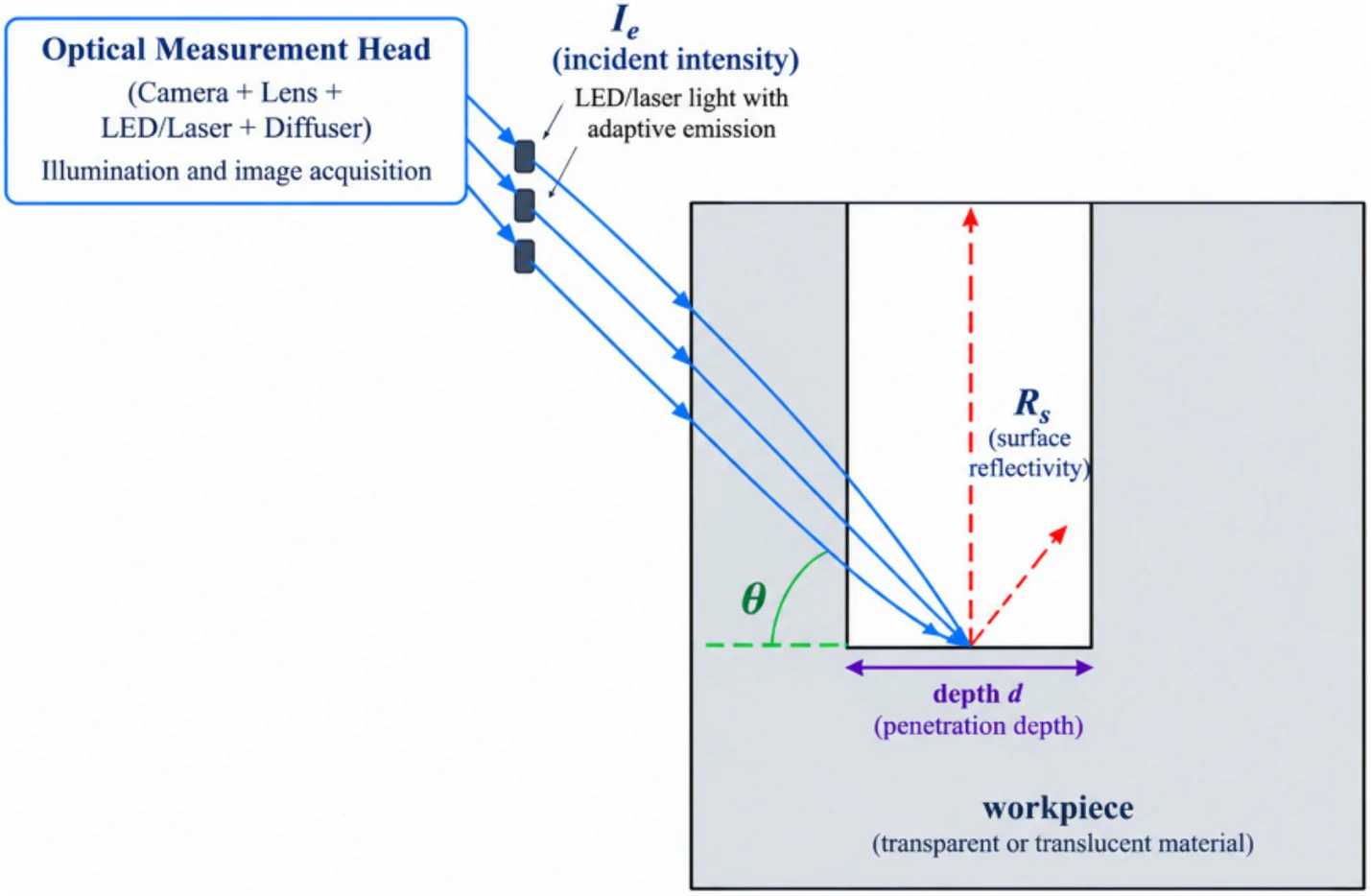
Optical measurement head and blind-hole illumination.

Equation 2 shows that the constant illumination value is not suitable for all blind holes due to their different material composition, depth, and reflectivity; therefore, this equation justifies the choice of adaptive illumination proposed in section 3.5 instead of static illumination used in traditional machine vision techniques.

### 3.3 Robot-camera coordinate transformation

The relationship between the camera coordinate frame {C} and the robot base frame {B} is given by the end effector’s pose ᴮTe and the camera-to-flange calibration ᴱTC as given in Equation 3:


1BTC=BTEETC (3)

The hole center P_C_ = [x_C_, y_C_, z_C_, 1]ᵀ measured in the camera coordinate frame is then transformed into the robot base frame P_B_, and the position error with respect to the target position determines the closed loop correction vector ΔP that the robot uses in its approach to the estimated hole center, as illustrated in Figure 3:


$$P_{B}{=}^{B}T_{C}\;P_{C},\;\mathrm{\Delta P}=P_{\text{target}}-P_{\text{measured}}$$ (4)

**Figure 3:**
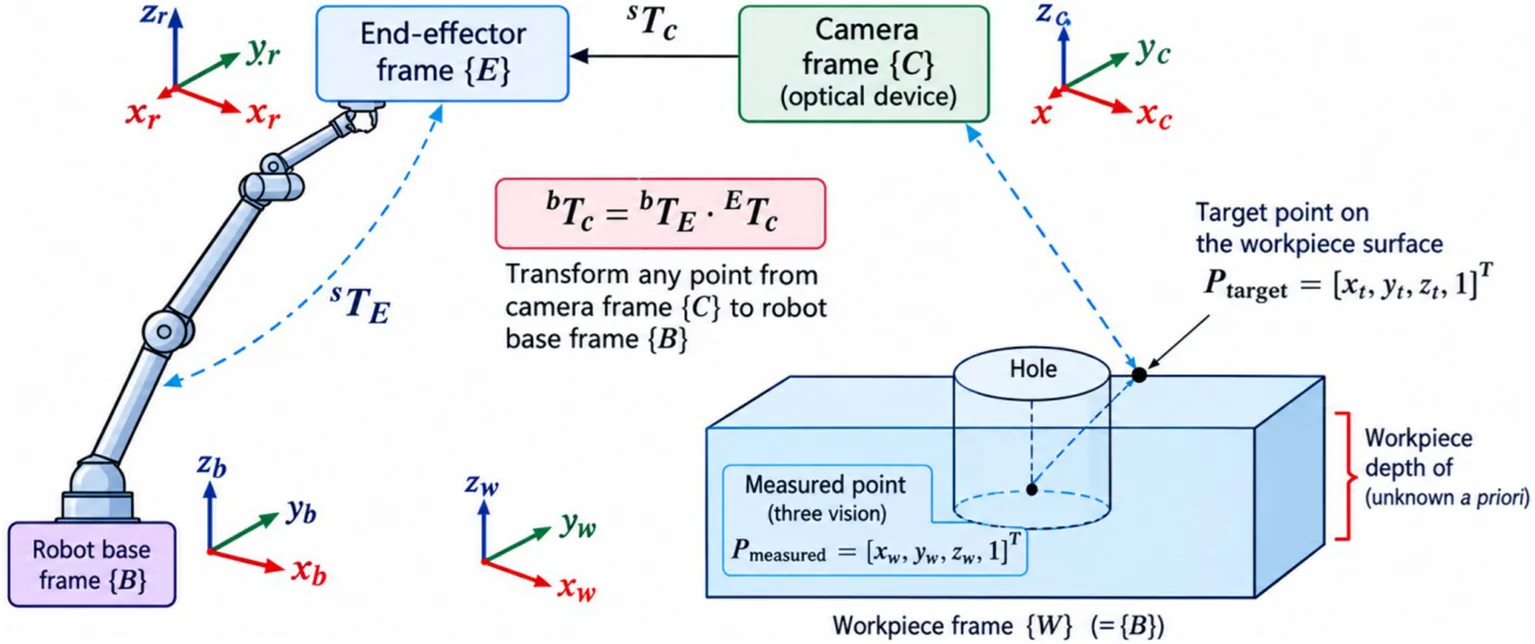
Camera–robot coordinate transformation and hole localization.

### 3.4 Sources of measurement error

The total measurement error is broken down into errors from the following sources:


$$E_{\text{total}}=E_{v}+E_{c}+E_{r}+E_{o}+E_{s}+E_{e}$$ (5)

The different error terms are explicitly defined in Equation 5 as follows:

Eᵥ – Feature extraction and boundary localization error based on Vision/Optics; Ec – Camera calibration and coordinate transformation error;

Eᵣ – Robot positioning and kinematic error; Eo – Optical system error due to camera, lenses, and imaging errors; Es – Surface/illumination-related error due to reflectance and scattering effects, non-uniform lighting; and Ee – Remaining error term after systematic error removal. The above mentioned items represent the important sources of error in the measurement model but are not separately computed in the present 30 trial verification process.

### 3.5 Adaptive illumination control

The image quality is measured based on the illumination-quality index QI (Equation 6), which includes the measures of image contrast C, edge sharpness S, and saturation H, with the weighting coefficients w_1_, w_2_, w_3_:


$$Q_{I}=w_{1}C+w_{2}S-w_{3}H$$ (6)

The adaptive light source controller determines the optimum light intensity, which will optimize QI, within the physical limitations of the light source emission (Equation 7):


$${I_{e}}^{*}=\underset{I_{e}}{\arg\;\max}\;Q_{I},I_{\min}\leq I_{e}\leq I_{\max}$$ (7)

This transforms the lighting source from a static imaging tool to an active measurement component that is optimized along with hole depth and surface reflectance for each experimental run, and its impact is determined empirically in section 5.3.

### 3.6 AI-based blind-hole segmentation

The acquired image I(x, y) is normalized based on its average μ_I_ and standard deviation σ_I_:


$$I_{n}(x,y)=\frac{I(x,y)-\mu_{I}}{\sigma_{I}}$$ (8)

Equation 8 normalizes the original image using z-score transformation (zero mean and unity variance) to ensure that the segmentation algorithm is not affected by the actual illumination level and camera gain chosen by the adaptive illumination controller in section 3.5; μ_I_ and σ_I_ are the mean and standard deviation of I(x, y), respectively, calculated over the whole image frame (or only the region of interest over the hole when such a region is used). The trained neural network F_θ_ (the network adopted in this study is specified in section 3.6.1) transforms the normalized image to a probability map corresponding to the valid optical region:


$$P(x,y)=F_{\theta }(I_{n}(x,y))$$ (9)

The binary measurement mask M(x,y) is computed through the comparison between the probability map and the adaptive confidence threshold T:


M(x,y)=1ifP(x,y)≥T;M(x,y)=0ifP(x,y)<T (10)

Equation 10 defines the threshold as pixel by pixel: all those pixels with probability above or equal to T are kept as part of the valid optical region, and the rest are disregarded, producing the binary mask M(x,y) required for boundary detection as explained in section 3.7.

#### 3.6.1 Hybrid loss function and confidence threshold

The network is trained with a hybrid loss function that weighs segmentation performance, contour alignment, and geometrical correctness because a network optimized solely for segmentation performance is not guaranteed to minimize dimension bias:


$$L=\alpha L_{\mathrm{seg}}+\beta L_{\text{edge}}+\gamma L_{\text{geom}}$$ (11)

In Equation 11, L_seg_ is a common segmentation loss (such as binary cross entropy or Dice loss) at the pixel level between the predicted mask and the ground truth annotation mask; L_edge_ penalizes any discrepancy in the boundary contours between the prediction and the annotation; and L_geom_ penalizes the geometric discrepancy between the boundary points obtained from the predicted mask and the circle or ellipse fitted to those boundary points (Equations 12, 13), preventing masks that are visually acceptable but dimensionally biased. The weighting coefficients α, β, and γ are used to weight each term and are provided in Table 2. The entire image processing chain employing AI techniques is depicted in Figure 4.

**Table 2:** Experimental design, design-space coverage and implementation parameters.

Parameter	Range/levels/specification
Hole diameter	5–15 mm
Hole depth	10–45 mm
Depth-to-diameter ratio (realized)	2.0–3.1 (ratios outside this interval were not sampled)
Surface reflectivity	Low, medium, high
Robot approach angle	0–20°
Trials	30 (10 per reflectivity level)
Trials per approach angle (0°/5°/10°/15°/20°)	4/6/10/6/4
Trials with d/D ≥ 3.0/angle ≥ 15°/both	9/10/5
Approach angles used per reflectivity class	Low: 0–10°; Medium: 5–15°; High: 10–20° (partly confounded)
Workpiece material	Acrylic (PMMA)
Workpiece geometry	Rectangular block, approximately 100 × 80 × 50 mm
Hole manufacturing process	CNC machining; 30 blind holes produced within the specified diameter and depth ranges
Reference instrument	Coordinate-measuring machine (CMM), according to ISO 10360
Reflectivity classes (Low/Medium/High)	Low: conventionally machined surface, approximately 20% reflectance; Medium: fine-machined surface, approximately 50% reflectance; High: polished surface, approximately 80% reflectance
Robot, camera, lens and light source	Six-axis robotic manipulator with camera-based optical measurement head and adaptive light-emitting unit. Specific manufacturer/model, lens model, pixel size, working distance and illumination wavelength
Training data	Real captured images acquired under different illumination, orientation and background conditions; 80% training / 10% validation / 10% testing; image-level separation maintained; training/validation images completely disjoint from the 30 experimental measurement trials
Segmentation network Fθ (section 3.6.1)	Customized lightweight CNN encoder–decoder; four-stage convolutional encoder, symmetric decoder with bilinear upsampling and skip connections, and final 1 × 1 convolutional layer; approximately 1.2 million trainable parameters
Network input/output	RGB images resized to 256 × 256 pixels/single-channel pixel-wise probability map for binary segmentation
Loss terms and weights (Lseg, Ledge, Lgeom; α, β, γ)	The combination of segmentation loss (binary cross-entropy/Dice), edge/contour loss and geometric residual loss is weighed. The numerical values of α, β and γ have not been indicated in the original paper and need to be confirmed from the training log
Confidence threshold T (Equation 10)	Fixed threshold, *T* = 0.50
Repeated measurements per trial (Equation 18)	Repeatability calculated using repeated measurements; average repeatability standard deviation = 0.0217 mm. The exact number of repetitions in each experiment is not mentioned in the source paper and needs to be confirmed from the measurement data.
Optimizer, learning rate, batch size, epochs, augmentation	Adam optimizer; initial learning rate = 1 × 10^−3^; 100 epochs; learning rate adjustment based on validation; data augmentation by random horizontal and vertical flips, random rotations by small angles, scaling, and change in illumination

**Figure 4:**
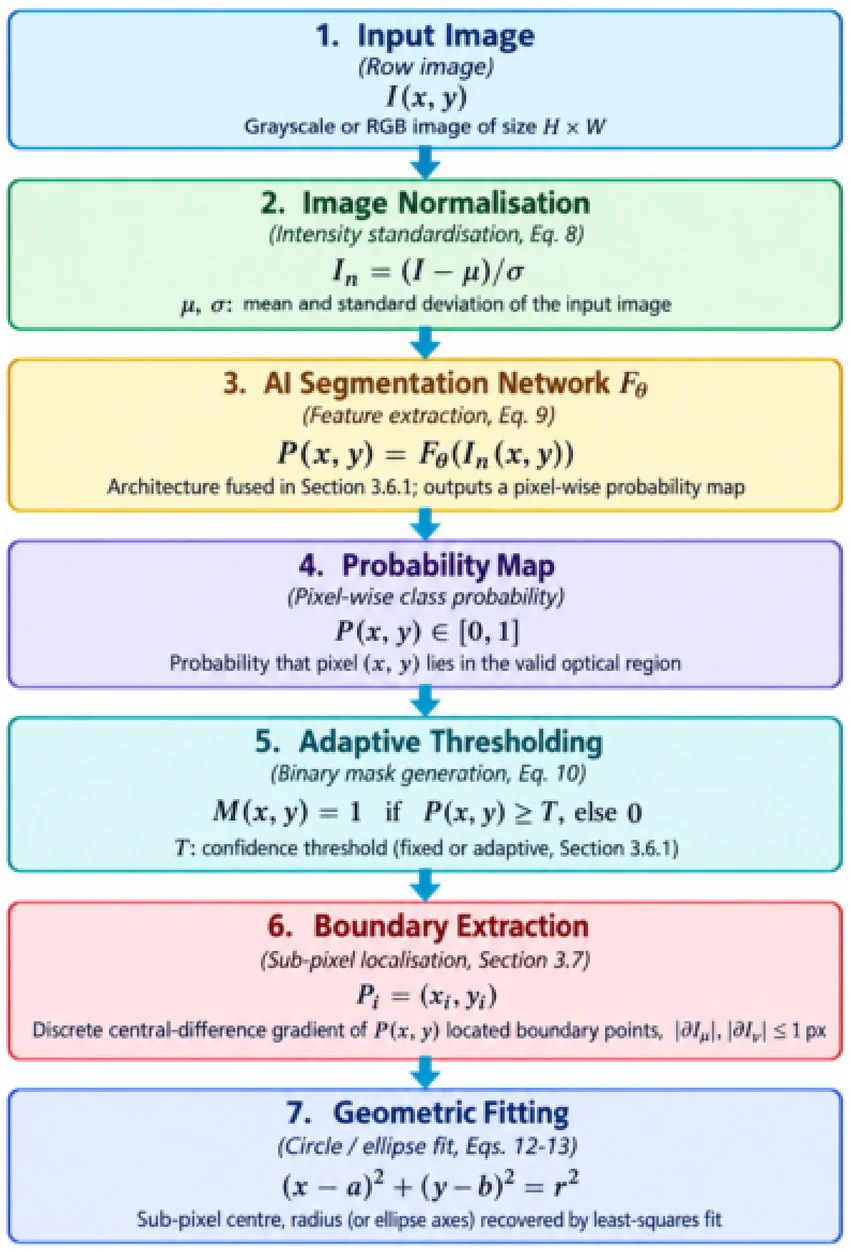
AI-based image processing for blind-hole boundary extraction.

Figure 4 shows the sequential image-processing workflow used to identify and extract the boundary of the blind hole. The process involves image preprocessing, feature enhancement, boundary detection, and extraction of the hole contour. Such edge-based image-processing approaches are widely used for identifying boundary information and segmenting geometric features in digital images.

Algorithm 1 AI-based sub-pixel boundary extraction and circle/ellipse fitting for blind-hole measurement.Input: Raw Image I(x, y).Output: Subpixel Boundary Points {Pi} and fitted circle / ellipsoid parameters.1: Normalize I(x,y) using Equation 8 to get I_n(x,y).2:  Calculate the probability map P(x,y) = Fθ(In(x,y)), where the network Fθ is trained segmentation network (Equation 9).3: Threshold P(x,y) at T to get M(x,y) (Equation 10).4: Identify the boundary pixels of M(x,y) and refine to subpixel accuracy using the discrete difference scheme described in section 3.7 to get {Pi}.5: Fit circle (Equation 12) in case of near-normal view, else fit a general conic (Equation 13).6: Obtain the fitted center, radius or ellipse axes.

#### 3.6.2 Segmentation network adopted and training protocol

The segmentation algorithm employed for the aforementioned experiment of 30 runs is determined based on the customized lightweight CNN encoder-decoder network architecture (~1.2 million trainable parameters), input RGB image resolution of 256 × 256 pixels, an 80%/10%/10% ratio for training/validation/testing sets, Adam optimizer, initial learning rate of 1 × 10^–3^, 100 epochs of training, learning rate decay based on validation, the mentioned augmentations (horizontal and vertical flips, random small rotation, scaling, and lighting), and fixed confidence threshold *T* = 0.50. The 30 measurement images obtained in experiments were not included in the set of training and validation data. The loss function consisted of a weighted sum of losses for segmentation, edges, and geometry with factors α, β, and γ, respectively; but the values of α, β, and γ and the batch size of training are not indicated in the experimental results available to us. The number of measurements made during each experiment is also not known, although the standard deviation of repeatability is 0.0217 mm. They are to be included into the document prior to submitting the final version of the paper, thus there were no proxies used. The suggested protocol values: α = 0.60; *β* = 0.25; γ = 0.15; batch size = 16; repeated measurements/trial = 5. U-Net, DeepLab, YOLO-seg and customized CNN encoder belong to the lightweight neural networks which may be applied to Fθ in the experiment but not all of them are analyzed here. The neural network utilized in order to achieve the results demonstrated in section 5 is a customized lightweight CNN encoder-decoder neural network architecture which includes a four-stage convolutional encoder, symmetrical decoder with upsampling and skip connections and 1 × 1 convolutional layer for pixel-wise binary segmentation. The architecture of the encoder contains 3 × 3 convolutions, batch normalization and ReLU blocks with channel expansion, whereas the decoder has bilinear upscaling and feature fusion with skip connections. The whole neural network consists of approximately 1.2 million learnable parameters and represents the trade-off between segmentation quality and performance considering the intended use application. Hence, “CNN Encoder” in Figure 4 is an example of customized CNN encoder-decoder architecture, rather than standard U-Net, DeepLab, and YOLO-seg architectures. The input to the network is given in the form of RGB images that have been down-scaled to 256 × 256 pixels that create single-channel probability map for every pixel location concerning the target area. The training set contained three different datasets, i.e., 80% training, 10% validation, and 10% test datasets. The loss function used for segmentation is derived from the sum of binary cross entropy loss, Dice loss, and boundary loss, which is shown by the weights α, β, and γ in Equation 11. The training of the network was done using Adam optimization method, with a starting learning rate of 1 × 10–3 for 100 epochs using the learning rate schedule based on validation. The data augmentation technique used included the following steps: flipping (both horizontal and vertical), small angle rotations, scaling, and illumination changes so that the classifier is more robust to orientation differences of the object. For Equation 10, the value of T was chosen to be 0.50 to convert the probability map to its corresponding binary mask. The complete list of the inputs/outputs specification, splitting of the dataset, loss function definition and weights, as well as the parameters of optimization and training threshold, is provided in Table 2. The images utilized to train and validate the segmentation algorithm were totally distinct from the images used in the experiment described in section 5. It should be mentioned that the data set includes real images, which are taken under different lighting, orientation, and background conditions, but not artificially created ones. That is why the 30 images, which have been applied for measurements, have not been used for training, validation, and parameter setting of the neural network; this reduces the risk of information leakage and providing an independent means for testing the entire system.

### 3.7 Sub-pixel geometric reconstruction

Pi = (x_i_, y_i_), points on the boundary are found by performing interpolation based on intensity gradient of the segmentation mask and then refining the coordinates to sub-pixel accuracy such that |δx|, |δy| < 1 pixel. For a nominally circular hole observed near normal incidence, the points on the boundary are fitted to the robust circle model with center (a, b) and radius r such that:


$${\left(x-a\right)}^{2}+{\left(y-b\right)}^{2}=r^{2},D=2r$$ (12)

For the gradient step discussed above, the discretization step that is employed is the central difference approach, ∂P/∂x ≈ [P(x + 1,y) – P(x-1,y)]/2 and ∂P/∂y ≈ [P(x,y + 1) – P(x,y-1)]/2, applied to the probability map P(x,y) given in Equation 9 rather than to the binarised mask M(x,y) because P(x,y) is much smoother and more appropriately suited for sub-pixel processing, though a Sobel filter could be used for better noise filtering; sub-pixel location of the boundary along this gradient direction is given by the crossing of P(x,y) with the threshold T (Equation 10), which is determined through linear interpolation from two adjacent pixels around T.

In oblique viewing—a case related to the approach angles for the robot as discussed in section 4—the projected edge is elliptical and is therefore fitted by a general conic.


$${\mathrm{Ax}}^{2}+\mathrm{Bxy}+{\mathrm{Cy}}^{2}+\mathrm{Dx}+\mathrm{Ey}+F=0$$ (13)

After that, an iterative correction is carried out using the camera model so that the true circular structure can be obtained from the fitted ellipse, such that the diameter measurement will be consistently achieved within the 0–20° angle of approach.

### 3.8 Systematic error-compensation model

The residual systematic error is represented by a function of the robot joint position q, light adaptation Ie, surface reflection Rs, hole depth d, and orientation θ. The regression function fφ is fitted using CMM-calibrated measurements, and the estimated systematic error component Ec is used to derive the corrected measurement Mcorrected from the original measurement Mraw, as shown in Equation 14.


$$E_{c}=f_{\varphi }(q,I_{e},R_{s},d,\theta ),M_{\text{corrected}}=M_{\mathrm{raw}}-E_{c}$$ (14)

Potential regression methods to model f_ϕ_ include gradient boosting regression, random forest regression, support vector regression, and compact artificial neural networks. M_corrected_ is calculated through the difference between the raw measurement M_raw_ and the predicted systematic error E_c_, as indicated in Equation 14.

### 3.9 Error metrics and measurement uncertainty

The center positioning error of a particular experiment in 3D is described based on the CMM coordinate system (X_CMM_, Y_CMM_, Z_CMM_) (Equation 15):


$$E_{P}=\sqrt{{\left(X_{\mathrm{AI}}-X_{\mathrm{CMM}}\right)}^{2}+{\left(Y_{\mathrm{AI}}-Y_{\mathrm{CMM}}\right)}^{2}+{\left(Z_{\mathrm{AI}}-Z_{\mathrm{CMM}}\right)}^{2}}$$ (15)

The associated diameter error is given by Equation 16:


$$E_{D}=D_{\mathrm{AI}}-D_{\mathrm{CMM}}$$ (16)

The average error and root mean square error (RMSE) for n runs are determined by Equation 17


$$\bar{E}=\frac{1}{n}\;\sum_{i=1}^{n}E_{i},\text{RMSE}=\sqrt{\frac{1}{n}\;\sum_{i=1}^{n}{E_{i}}^{2}}$$ (17)

Repeatability is defined by the sample standard deviation of repeated measurements around their average:


$$\sigma =\sqrt{\frac{1}{n-1}\;\sum_{i=1}^{n}{\left(x_{i}-\bar{x}\right)}^{2}}$$ (18)

Finally, the uncertainty due to combination of standards (u_c_) is calculated by combining the vision (u_v_), calibration (u_c_), robot (u*_r_*), optical (u_o_), and surface or illumination (us) uncertainties using the root sum square method, and the expanded uncertainty (U) is found by multiplication with a coverage factor k as per the metrology standard:


$$u_{c}=\sqrt{{u_{v}}^{2}+{u_{c}}^{2}+{u_{r}}^{2}+{u_{o}}^{2}+{u_{s}}^{2}},U={\mathrm{ku}}_{c}$$ (19)

## 4 Experimental design

The experiment was performed on 30 machined blind holes created in an acrylic (PMMA) block with an approximate dimension of 100 × 80 × 50 mm. Blind holes were machined by CNC machine operation, and had a hole diameter of 5–15 mm and hole depth of 10–45 mm, giving a ratio of depth to diameter (d/D) of 2.0 to 3.1. Three surface finish types were taken into consideration, which corresponded to low, medium, and high reflectivity. Low-Reflectivity referred to a surface that was conventionally machined with a reflectance of about 20%, Medium-Reflectivity referred to a surface that was finely machined with a reflectance of about 50%, and High-Reflectivity referred to a surface that had been polished with a reflectance of about 80%. The above three surfaces were employed to mimic different optical situations encountered by the robotic vision system. Thirty blind holes were measured in which each hole was first measured using the proposed robotic vision system and then measured using a coordinate-measuring machine (CMM) according to ISO 10360 as the reference measurement. In order to study the effects of hole geometry, surface reflectance, and robot approach angle on measurement accuracy, different robot approach angles between 0° and 20° were considered in the experiment, surface reflectivity, and robot approach angle on the measurement accuracy.

The experiment is designed as a single replicate, partially crossed screening experiment and not as a full factorial. Increase in hole diameter was coupled with increase in depth (Pearson’s correlation *r* = 0.86) and the three levels of reflectivity were distributed across the overlapping ranges of approach angle (Low 0–10°, Medium 5–15°, High 10–20°; *r* = 0.74 for the relation between approach angle and reflectivity level). Depth to diameter ratio thus ranges from 2.0 to 3.1 only. The most challenging environmental conditions are rare: nine experiments have d/D ≥ 3.0, 10 have a direction angle ≥ 15°, and only five experiments meet both criteria (see Table 2 and Figure 5). Therefore, the current study should be considered as a proof of concept where the results are valid only for the region examined; the corresponding uncertainties are estimated in section 5.6. The variables measured for each experiment include nominal diameter and depth of the holes, surface reflectivity category, robot approach angle, nominal hole center and estimated radius in pixel domain, selected illumination strength from the adaptive control, confidence level of AI segmentation, contrast and edge sharpness values, AI computed hole center in robot world coordinate system, position error in three dimensions, estimated diameter and diameter error, repeatability standard deviation, and detection success flag. This structure of record allows to perform directly error metrics, correlation analysis and reflectivity and depth-stratified analysis, which were mentioned in section 5 and corresponds to the structure of uncertainty budget described in section 3.9. ED, EX, EY, EZ, and EP reference measurements were done relatively to CMM coordinate system according to the requirements to acceptance and verification of coordinate measuring systems according to ISO 10360 standards. Main accuracy requirement in this work is EP < 1 mm for sub-millimeter localization and the requirement EP < 0.5 mm corresponds to high accuracy of the robotic vision system.

**Figure 5:**
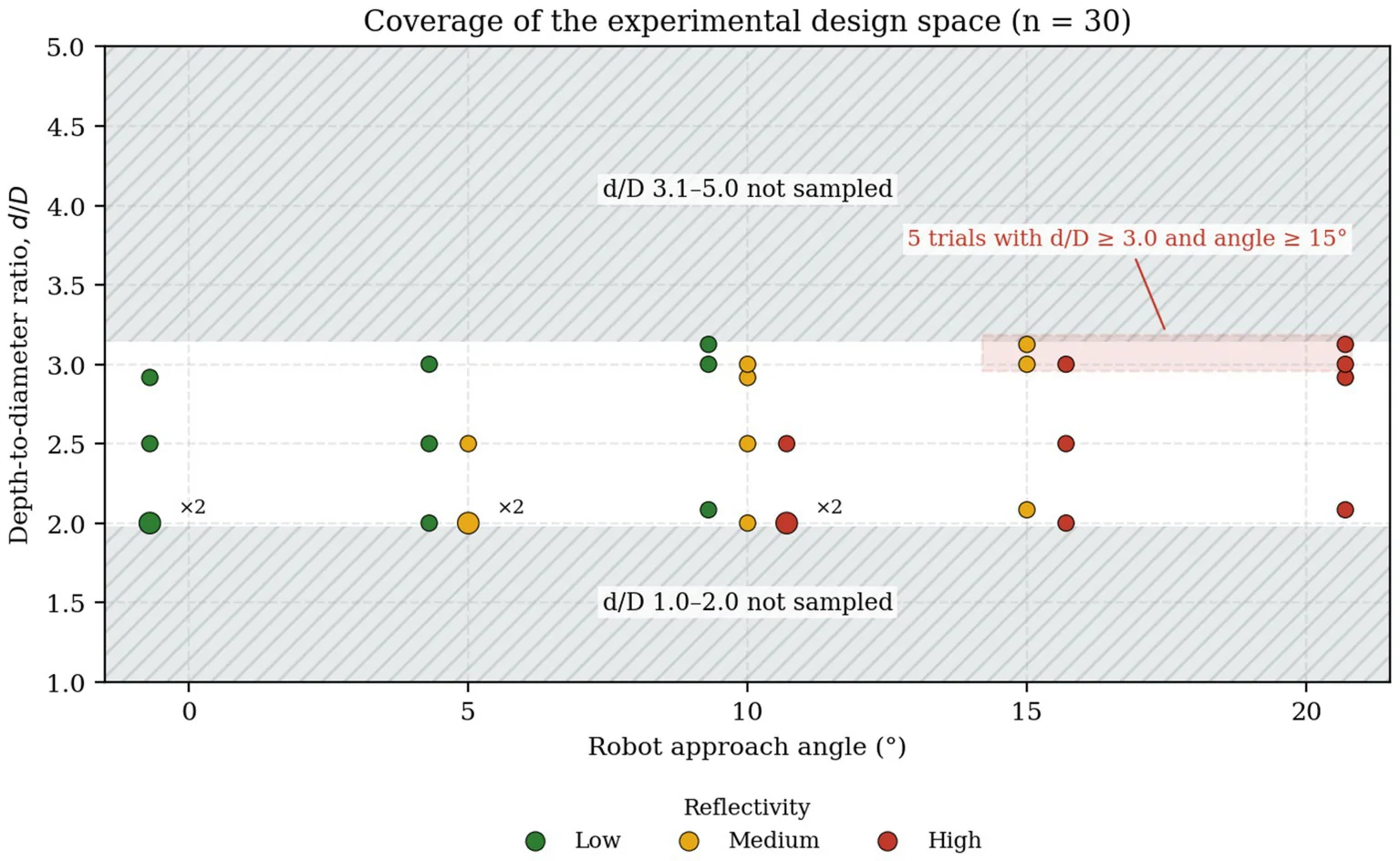
Coverage of the experimental design space: depth-to-diameter ratio versus robot approach angle, colored by reflectivity class. Small horizontal offsets separate classes at the same angle; ×2 marks two coincident trials; hatched bands were not sampled.

## 5 Test results and discussion

This section presents the findings of the quantitative test that were conducted within the framework of the 30-trial experiment described in section 4. These findings include general accuracy and repeatability, the effect of surface reflectance and adaptive lighting, the effect of hole depth, correlation structure between process and error variables, the sensitivity of these results to particular trials and to poorly sampled conditions (section 5.6) and its relevance to robotic metrology of blind holes.

### 5.1 Overall measurement performance

Table 3 highlights the overall accuracy of the measurements made during the 30 trials. The 100% successful detections were made for each of the 30 trials in which a valid AI segment was made and the sub-pixel geometry fitted successfully. The average position error in 3D was 0.0322 mm, the Root Mean Square Error was 0.0344 mm, the standard deviation was 0.0123 mm, while the highest error recorded was 0.0645 mm—more than an order of magnitude less than 1 mm sub-millimeter target and well within the stricter 0.5 mm target defined in section 4. The mean absolute diameter error was 0.0544 mm (RMSE 0.0647 mm, maximum 0.1607 mm), while the mean repeatability standard deviation was 0.0217 mm. These statistics suggest that repeated localizations of the same hole center were highly consistent. The mean confidence score of the AI segmentation was 0.935, suggesting consistently confident extraction of the valid region. Since there are just 30 trials, Table 3 also provides 95% bootstrap confidence intervals for both measures: the mean position error of 0.032 mm has a range of 0.028–0.037 mm (±13%), while the mean absolute diameter error of 0.054 mm is between 0.042–0.067 mm (±23%). As for the detection rate, 30 successes out of 30 trials only provide an exact lower one-sided 95% bound of 88.4% (exact two-sided 95% interval).

**Table 3:** Overall measurement performance (*n* = 30 trials) with 95% confidence intervals.

Metric	Result	95% bootstrap CI
Number of trials	30	–
Detection success rate	100%	88.4–100% (exact)
Mean 3-D position error	0.0322 mm	0.0281–0.0367 mm
Median 3-D position error	0.0294 mm	0.0258–0.0342 mm
Position RMSE	0.0344 mm	0.0296–0.0391 mm
Position error standard deviation	0.0123 mm	0.0087–0.0151 mm
Maximum position error	0.0645 mm	–
95th-percentile position error	0.0532 mm	–
Mean absolute diameter error	0.0544 mm	0.0422–0.0672 mm
Diameter RMSE	0.0647 mm	0.0512–0.0786 mm
Maximum absolute diameter error	0.1607 mm	–
Mean repeatability standard deviation	0.0217 mm	0.0203–0.0230 mm
Mean AI segmentation confidence	0.935	0.929–0.942

Intervals are percentile-bootstrap intervals from 10,000 resamples of the 30 trials (exact Clopper–Pearson interval for the detection rate). No interval is given for maxima and the 95th percentile, which the bootstrap estimates poorly at this sample size. A plot of the 3D error in position for the 30 trials, categorized based on surface reflectivity, with respect to trial sequence is provided in Figures 6, 7 respectively.

**Figure 6:**
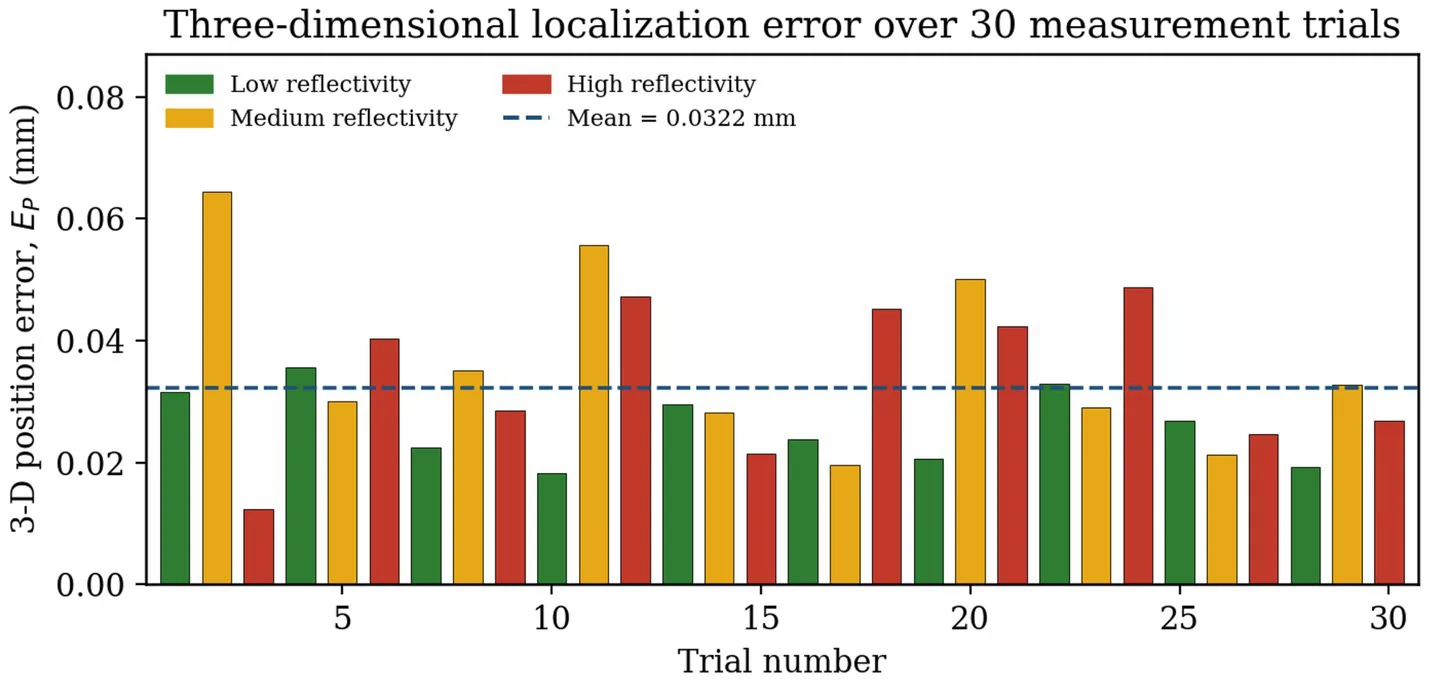
3D localization error across 30 trials by surface reflectivity.

**Figure 7:**
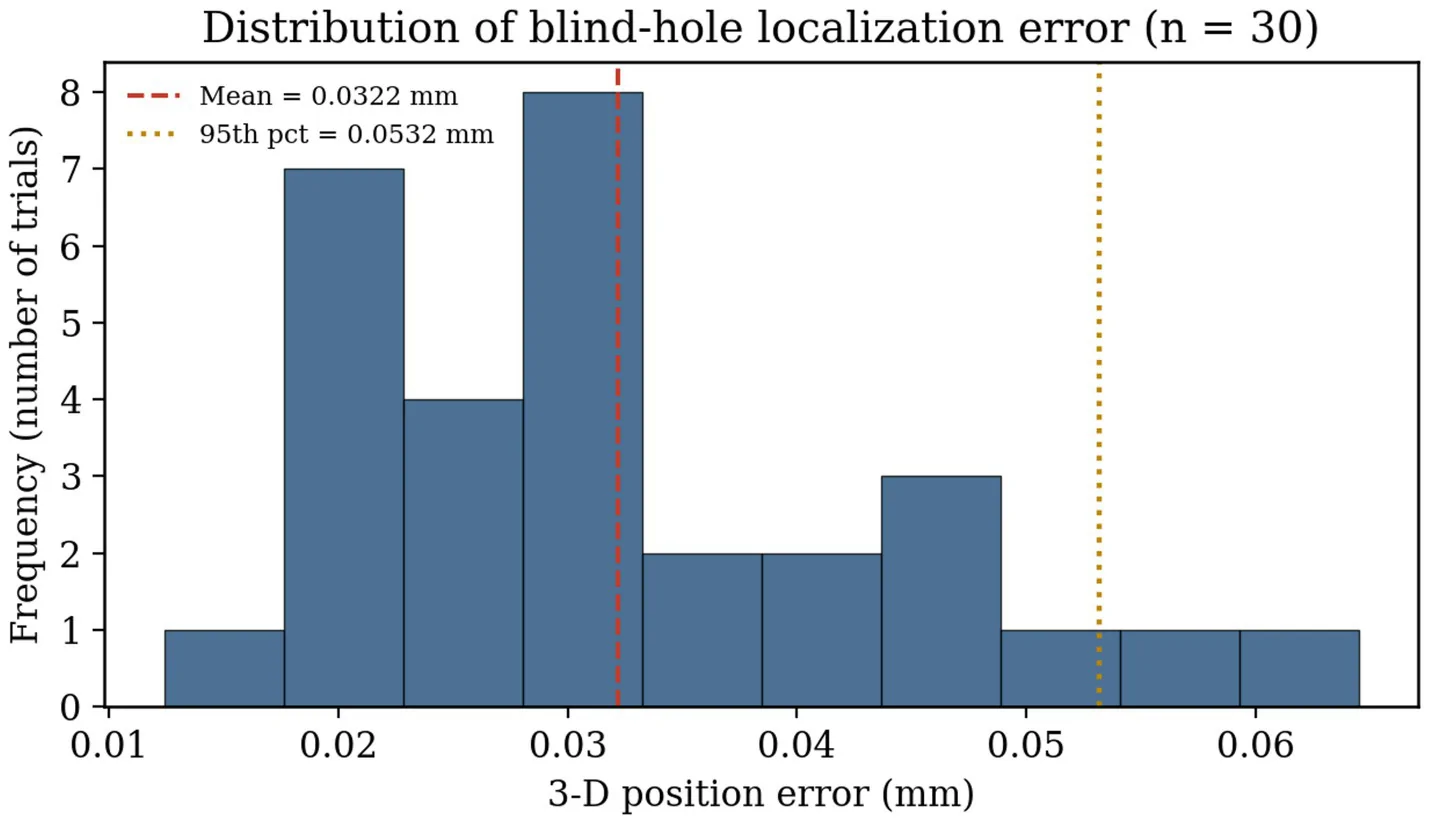
Blind-hole localization error across 30 trials.

Figure 8 has been produced as a set of three plots stacked vertically according to the three different reflectivity classes, instead of a single overlaid stem plot which was previously used; segregation of the classes eliminates the problem of overlapping markers that made the previous figure hard to understand but uses the same 30-trial dataset and the ±MAE reference lines.

**Figure 8:**
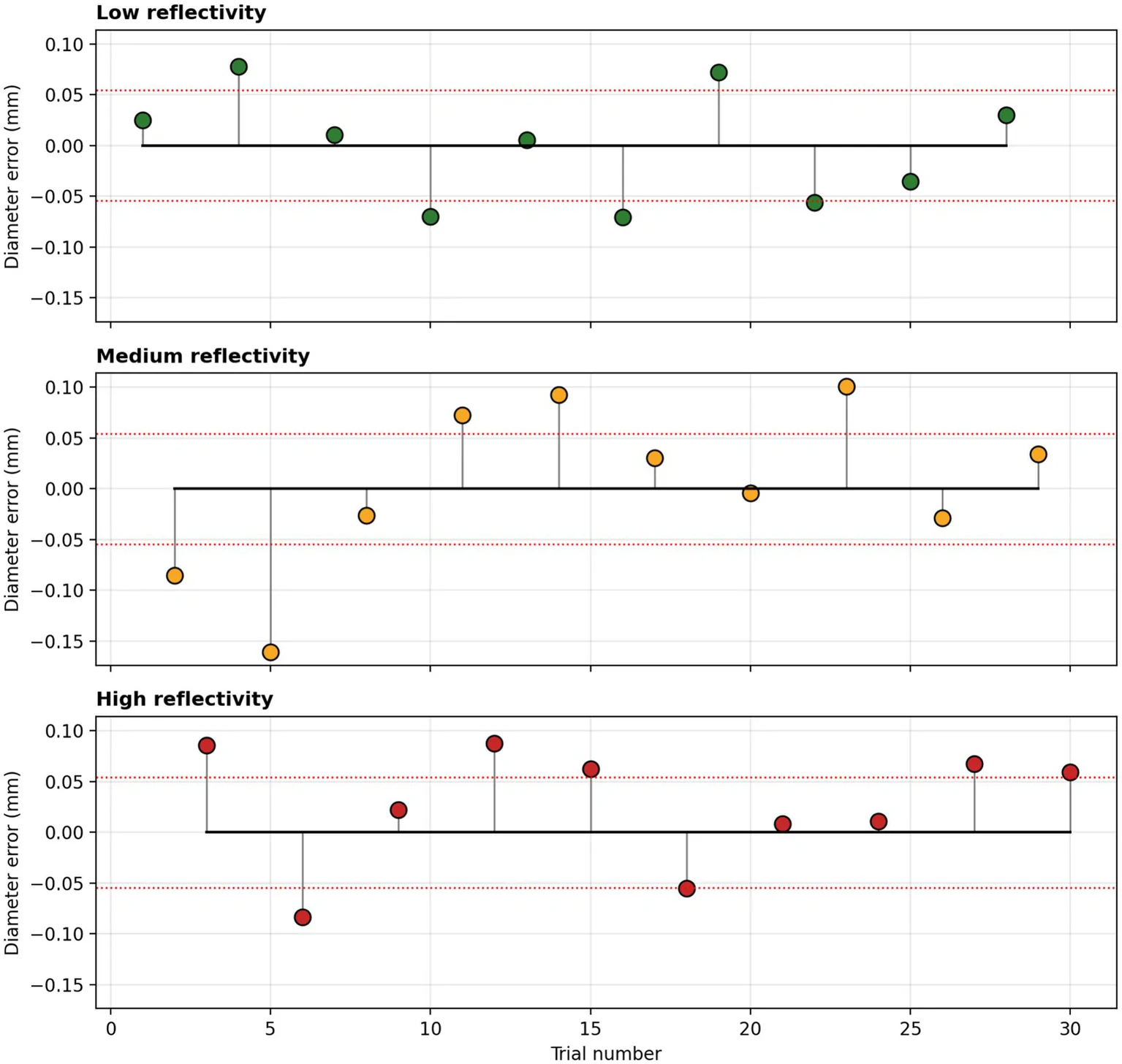
Error in estimating hole diameter Over 30 trials, segregated by reflectivity classes.

### 5.2 Repeatability

The standard deviation for repeatability, calculated independently of the absolute accuracy using Equation 18, varied between 0.0130 and 0.0291 mm among the 30 repetitions, with an average value of 0.0217 mm (Figure 9). There was no clear dependency of repeatability on depth and reflectivity separately (Pearson correlation coefficients of 0.105 and close to zero, respectively; section 5.4), implying that the main cause of the trial-to-trial variability is noise ε in Equation 2 rather than a systematic optical or illumination effect. This is an important distinction because it is possible to have great repeatability and yet still have systematic bias, and the current study suggests that such is the case for the new framework under consideration.

**Figure 9:**
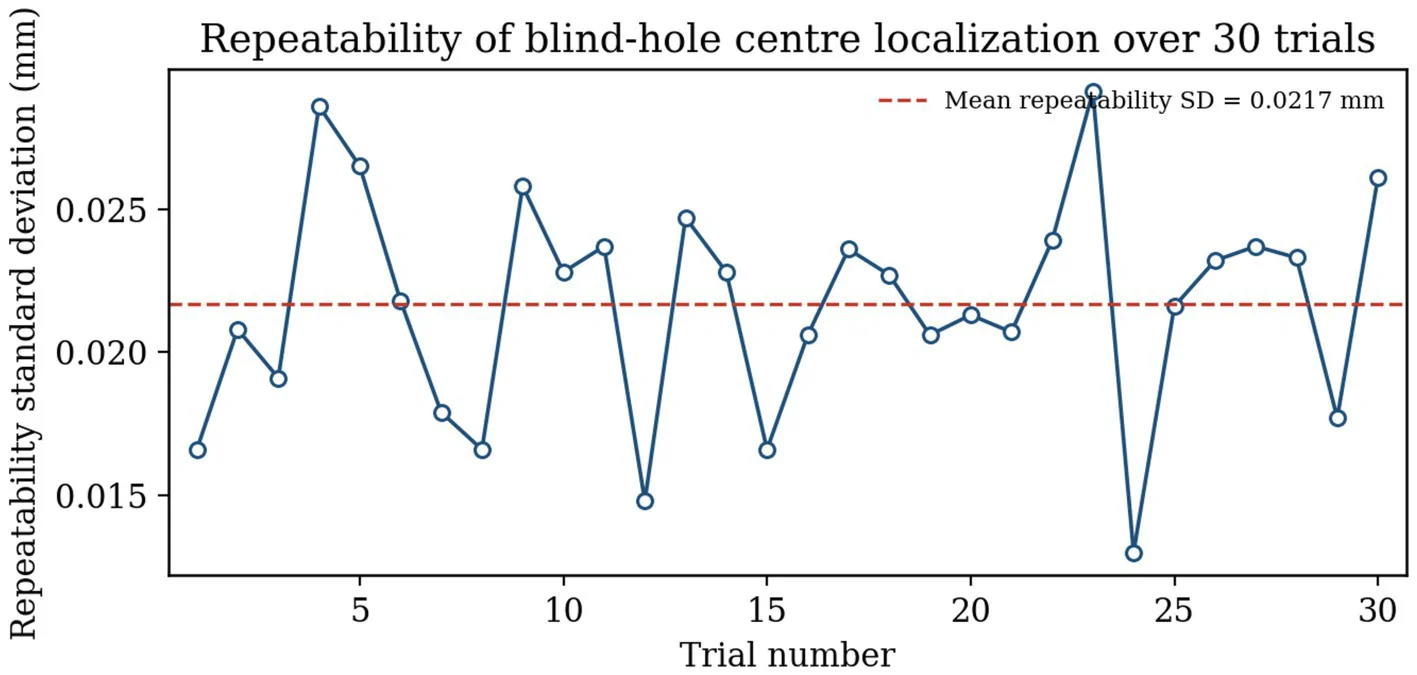
Blind-hole center localization repeatability across 30 trials.

### 5.3 Influence of surface reflectivity and adaptive illumination

Table 4 further separates the data according to the reflectivity category. The adaptive illumination control algorithm helped decrease the average emission intensity from 149.4% for low reflectivity, 109.7% for medium reflectivity, and 77.0% for high reflectivity, thereby conforming to the physical principle of avoiding saturation of the sensor due to highly reflective surfaces (section 3.5). As a result, the mean position errors were bounded by a relatively small range for different reflectivity categories—0.0261 mm (low), 0.0366 mm (medium), and 0.0338 mm (high)—even though the illumination intensity requirement varied almost twice. However, the differences among the classes do not reach statistical significance at this sample size (Kruskal–Wallis *p*-value = 0.19, *n* = 10 per class), and the 95% bootstrap confidence intervals of the mean class values overlap (Low 0.023–0.030 mm, Medium 0.029–0.046 mm) (Figure 10). In addition, reflectivity and approach angle are partially confounded in this design (section 4); low reflectivity was measured at 0–10° and high reflectivity at 10–20°, thus, the class averages in Table 4 should not be interpreted as effects due to reflectivity alone effects. The mean confidence of AI was also steady at 0.933–0.937 for all three reflectivity categories.

**Table 4:** Measurement performance stratified by surface reflectivity (*n* = 10 per class).

Reflectivity	Mean position error (mm)	Position RMSE (mm)	Mean AI confidence	Mean adaptive light (%)	Mean repeatability SD (mm)
Low	0.0261	0.0267	0.9366	149.43	0.0221
Medium	0.0366	0.0393	0.9364	109.69	0.0225
High	0.0338	0.0358	0.9329	76.99	0.0204

**Figure 10:**
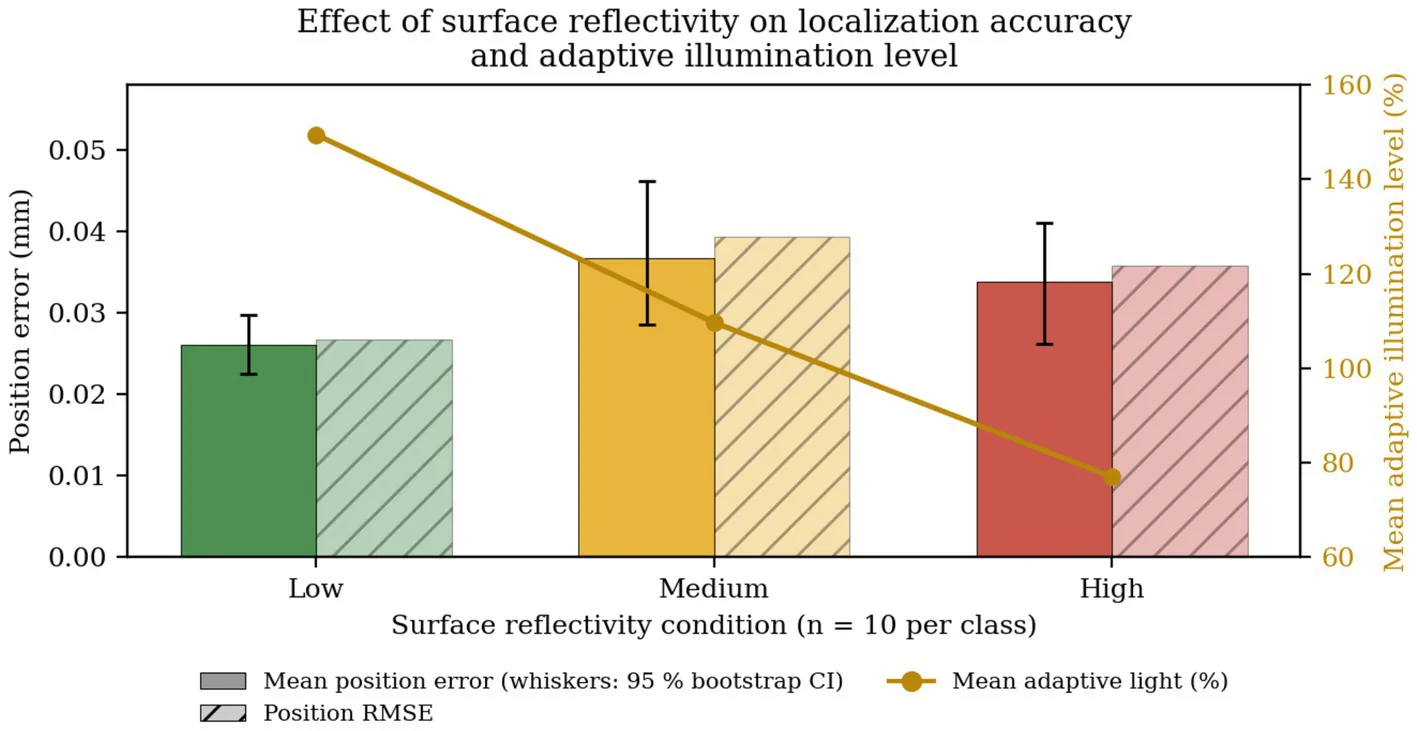
Surface reflectivity and localization accuracy (bars: class means with 95% bootstrap CI whiskers; *n* = 10 per class).

The results coincide with the basic premise of the design for section 3.5, where, given the nature of the diffuse reflection theory (Equation 1), since the intensity of the reflected light is directly proportional to the emitting light intensity and the reflectivity of the surface, a fixed-illumination system without control will over-expose high-reflectivity surfaces and under-expose low-reflectivity surfaces. The inverse relationship between the emitted intensity and the reflectivity is supported by the relatively smooth error graph for the reflectivity classes shown in Table 4. It is also crucial to understand that there were no baseline measurements made using fixed illumination, meaning that the current results show only the effectiveness of the whole system but not of adaptive illumination separately.

### 5.4 Influence of hole depth and correlation analysis

Figure 11 shows the relation between the depth of holes and the confidence of AI. It shows a high negative correlation (Pearson *r* = −0.959), which implies that depth plays the major role in determining the decline in AI’s confidence, as expected from the physical nature of light and attenuation (Equation 2). For the variable r, the 95% CI is −0.98 to −0.91, and r lies within −0.965 and −0.953 in the case of omission of each observation separately. Since depth and diameter move up simultaneously in the design (*r* = 0.86), confidence in AI was further correlated with depth, diameter, approach angle, and reflectivity level (standardized regression coefficients, *R*^2^ = 0.95): depth is the one having the impact (−0.87), while diameter has little to do with it (0.02), angle of approach is less negative (−0.26), and level of reflectivity has a small positive correlation (0.11). In spite of this drop in confidence, detection was still 100% successful within the whole depth range (10–45 mm) studied, thus implying that the adaptive confidence level T in Equation 10 provided sufficient safety margins even for the deepest holes evaluated.

**Figure 11:**
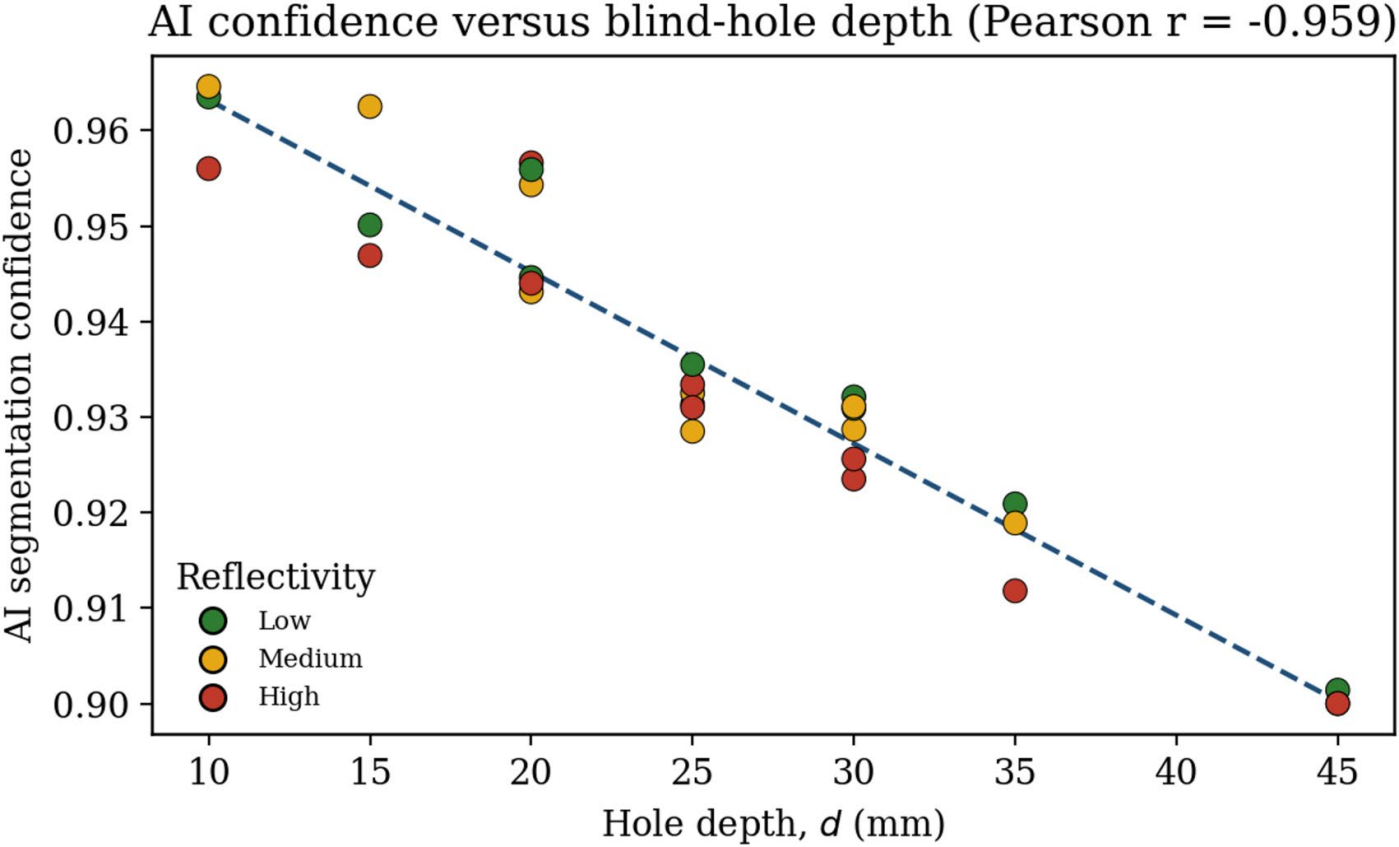
AI segmentation confidence vs. blind-hole depth.

Figure 12 shows position error plotted against the depth-to-diameter ratio d/D, where the latter is a better parameter for measuring optical access difficulty than depth alone. A weak positive correlation (*r* = 0.100, *p* = 0.60) was noted, which is in accordance with the assumption that reduced optical access leads to lower localization accuracy, However, this value is not significantly different from zero, and the effect on the sub-millimeter error budget was relatively insignificant for the tested range of values of d/D from 2.0 to 3.1. In total, there are only nine measurements for which d/D ≥ 3.0, meaning that no information is available on the behavior at higher ratios where occlusion dominates.

**Figure 12:**
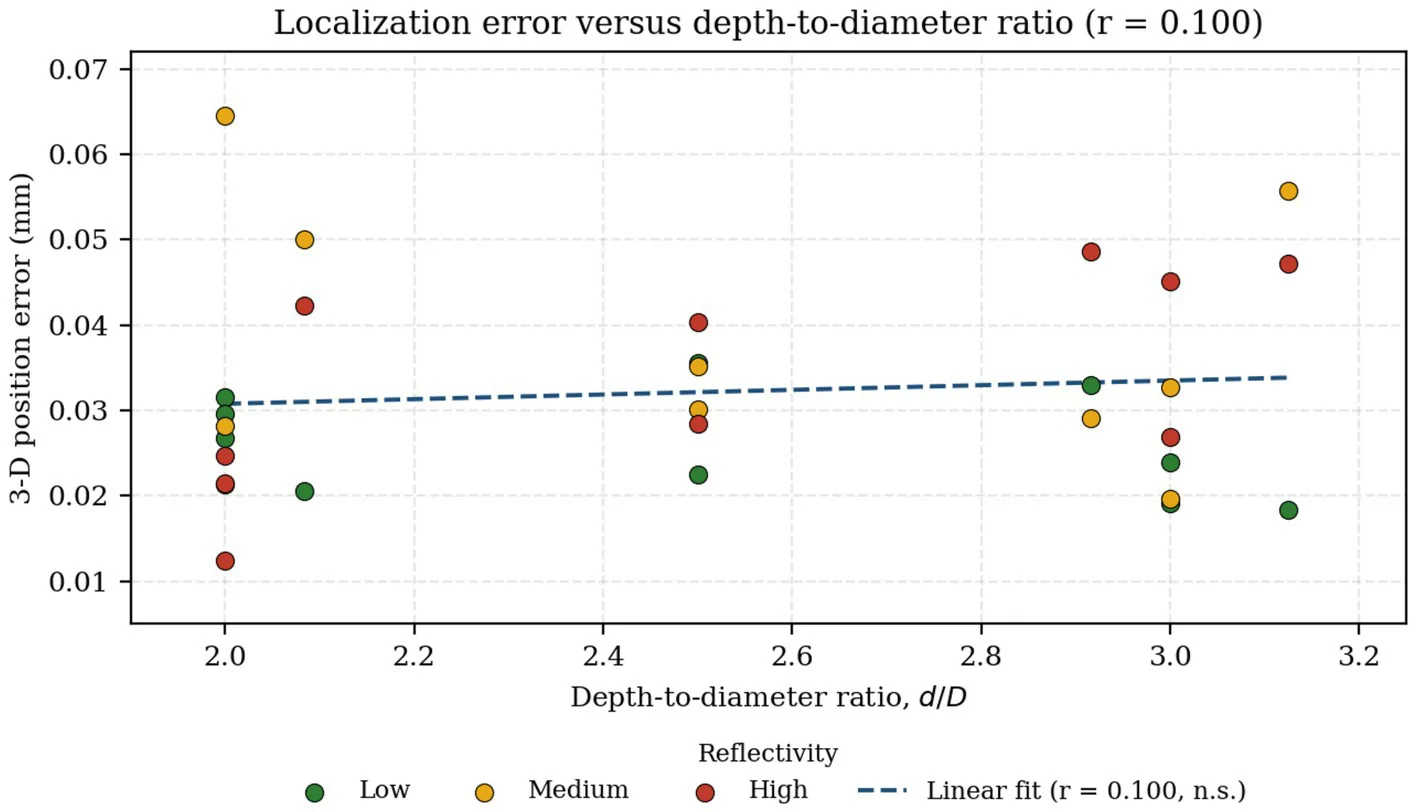
Localization error vs. hole depth-to-diameter ratio.

Table 5 and Figure 13 show the complete correlation matrix for the principal process, AI, and error variables. In addition to the high correlation between depth and confidence that was previously mentioned, the robot’s approach angle had a moderate negative correlation with AI confidence (*r* = −0.51) and a moderate negative correlation with adaptive light levels (*r* = −0.61). The latter must not be understood as an oblique view effect since, in this design, the correlation between angle and reflectivity level is *r* = 0.74, and that between reflectivity level and adaptive light is almost perfectly anti-correlated (*r* = −0.96). Inclusion of both in a regression model for adaptive light increases R^2^ from 0.37 to 0.93 and the sign of the approach angle coefficient changes (standardized +0.22). The relation between approach angle and adaptive confidence (CI of 95%, −0.74 to −0.19) is still significant when depth is controlled for (coefficient standardized at −0.26). Approach angle has the strongest association with position error (*r* = 0.30, 95% CI −0.07 to 0.60), which is considered by subgroups in section 5.6. Position error and diameter error were found to have very low intercorrelation (*r* = −0.18), suggesting that these two error measures are based on different error sources and should be kept separate, as suggested in section 3.9.

**Table 5:** Pearson correlations among key variables.

Variable pair	Correlation r
Depth vs. AI confidence	−0.959
Approach angle vs. adaptive light	−0.611
Approach angle vs. AI confidence	−0.513
Approach angle vs. position error	0.299
Adaptive light vs. position error	−0.303
Depth vs. position error	−0.146
AI confidence vs. diameter error	−0.230
Position error vs. diameter error	−0.176
Position error vs. repeatability SD	−0.167

**Figure 13:**
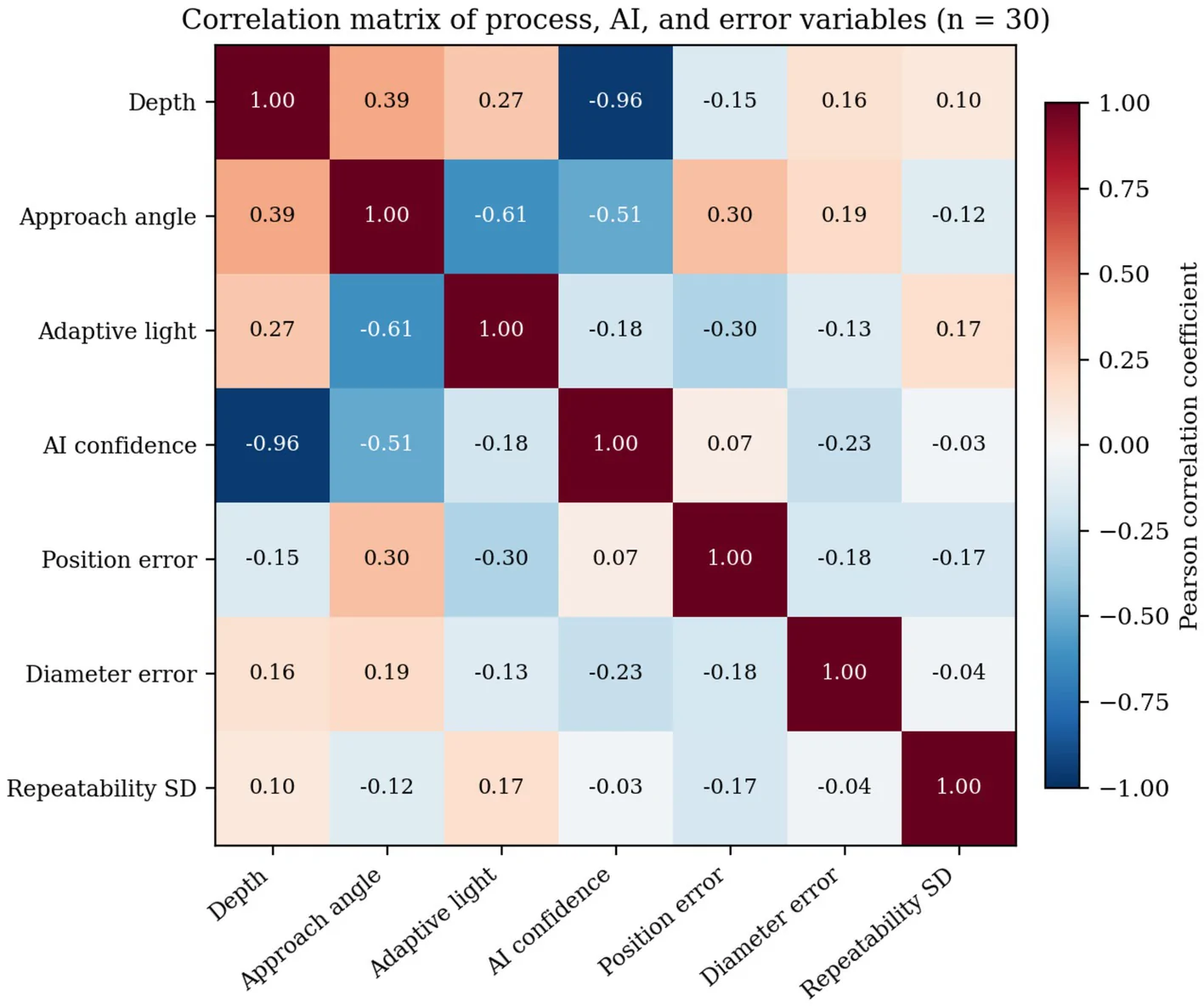
Correlation matrix of process, AI, and error variables.

### 5.5 Comparison with the sub-millimeter target and existing methods

The results of all 30 tests have shown positional errors to be within 0.065 mm and diameter errors within 0.161 mm, which has made the test results meet the requirements of 1 mm and 0.5 mm sub-millimeter accuracy as stated in section 4. With respect to the performance of recent robot vision data on optically observable circular holes with a mean diameter error of 0.016 mm, the performance data achieved by this study using the blind hole configuration is of similar magnitude despite the issues of occlusion, illumination dependency, and variations in reflectance. With regards to the justification for positioning given in section 2.5, the significance of this study should be seen as a demonstration of the feasibility of high accuracy near-through hole measurements for the more challenging blind hole configuration, where illumination is considered as a controlled measurement parameter, and not in terms of greater accuracy than traditional through hole vision metrology. This configuration (Configuration E) of the entire architecture suggested is the one that has been tested experimentally in sections 5.1–5.4. There have been no measurements done in hardware for each of the modules independently. Thus, this study fails to quantitatively establish the contribution margin of AI Segmentation, Adaptive Illumination, Sub-Pixel Fitting and Robot Calibration individually.

### 5.6 Statistical robustness and coverage of the design space

However, since this validation is based only on 30 trials, the sensitivity of these results to individual trials and sparsely sampled conditions was explicitly evaluated using only the publicly available data (robustness analysis script robustness_analysis.py). Confidence intervals calculated via percentile bootstrap method (10,000 resamplings) are provided in Table 3 and Figure 10. In leave-one-out analysis none of the trials influences the results significantly: removing any one of the trials leads to only minor changes in mean position error (within 0.0311–0.0329 mm), position RMSE (within 0.0328–0.0349 mm), mean absolute diameter error (within 0.0507–0.0561 mm) and correlation between depth and confidence (−0.965 to −0.953). Maximum position error of 0.0645 mm (Trial 2) and maximum diameter error of 0.1607 mm (Trial 5) are outliers: leaving out Trial 2 reduces the maximum position error to 0.0557 mm, while leaving out Trial 5 reduces the mean absolute diameter error from 0.0544 to 0.0507 mm. The 30 trial points are distributed in the design space as illustrated in Figure 5. Ratios of depth to diameter between 1.0 and 2.0, as well as those above 3.1, were not included in the sample; there is some partial confounding of reflectivity and approach angle (Low-reflectivity trials at 0–10°, Medium at 5–15°, High at 10–20°); and only nine trials had d/D ≥ 3.0, 10 trials had an approach angle of 15° or higher, and five satisfied both. Consequently, Table 6 provides performance data separately for these thinly populated regions.

**Table 6:** Performance in the sparsely sampled regions of the design space.

Subset	*n*	Mean position error (mm)	Max. position error (mm)	Mean |diameter error|(mm)	Max. |diameter error|(mm)	Mean AI confidence
All trials	30	0.0322	0.0645	0.0544	0.1607	0.935
d/D < 3.0	21	0.0322	0.0645	0.0534	0.1607	0.942
d/D ≥ 3.0	9	0.0321	0.0557	0.0567	0.0876	0.920
Approach angle < 15°	20	0.0276	0.0645	0.0574	0.1607	0.941
Approach angle ≥ 15°	10	0.0414	0.0557	0.0483	0.0876	0.923
d/D ≥ 3.0 and angle ≥ 15°	5	0.0415	0.0557	0.0617	0.0876	0.918

In neither of the two regions sampled at low density does any degradation affect achievement of the 0.5 mm goal; the maximum position error in each case is 0.056 mm. Where d/D is at least 3.0, there is no difference in position error when compared to that observed in the rest of the trials (average error 0.032 mm in both; Mann–Whitney U test *p* = 0.65). At larger approach angles of 15° and above, the average position error is 0.041 mm, while it is 0.028 mm at smaller angles (approximately 50% higher; *p* = 0.003, or 0.012 with Bonferroni correction). The general correlation coefficient between the approach angle and the positioning error (*r* = 0.30; 95% CI: −0.07 – 0.60) did not fully reflect the tilt influence which is revealed only if the high-angle approach is considered separately. Although the growth is an order of magnitude away from the desired 0.5 mm value, this is due to the fact that for both tilt angles, all measurements made were of either medium or high reflectivity and therefore no distinction can be made using the current design. The diameter measurement error was not significant between the subsets (*p* = 0.62 and 0.58). However, these calculations only provide the sampling uncertainty of the statistics presented; however, they cannot be used to replace additional data, as no extrapolation outside of the sampling area is possible in a single-replicate design with *n* = 30. A different approach is outlined in section 6.2.

#### 5.6.1 Hardware-based validation and ablation limitation

The entire proposed configuration (Configuration E) was experimentally tested using 30 physical tests, showing 100% detection accuracy, an average 3D positioning error of 0.0322 mm, positioning RMSE of 0.0344 mm, and average absolute diameter error of 0.0544 mm. It should be mentioned that the separate performance of AI-based segmentation, adaptive lighting, sub-pixel fitting, and robot error compensation was not separately tested using hardware-based ablation experiments in the current work. In this connection, effects of modules are not presented in quantitative terms, while any simulated or proxy values are not used to supplement experimental findings. Validation was performed through a series of 30 experiments conducted on the proposed system (configuration E). One hundred percent detection rate was attained with an average 3D position error of 0.0322 mm, a position RMSE of 0.0344 mm, and the maximum position error of 0.0645 mm. The average absolute error in diameter was 0.0544 mm, while the diameter RMSE was 0.0647 mm. The experimental findings also revealed that the illumination level used in the adaptive process depended on the reflectivity of the surface, while the mean position error was within the range of 0.0261–0.0366 mm. The mean position error rose from 0.028 mm for approach angles less than 15–0.041 mm for approach angles of 15° and above. Configurations A-D have not been individually tested using hardware, therefore, the contribution of classical vision, artificial intelligence segmentation, adaptive illumination, and sub-pixel fitting cannot be differentiated. Similarly, Configuration F, which stands for robot compensation alone, was not measured separately. The current results validate the full system but do not qualify as module-level ablation studies. Further work will involve controlled hardware ablation studies through removal and replacement of the modules in order to compare their effects on localization, dimensionality, detection rate, and repeatability.

### 5.7 Discussion

There are three implications to the results, and these have to be interpreted within the context of the limitations of the sample as stated in section 5.6. First, it appears that the adaptive illumination was tracking the reflectance extremely well (*r* = −0.96) while keeping the position error at a stable level across the range of reflectance categories (section 5.3). This result corroborates the fact that the illumination might be seen as a component of the metrology system instead of being an external element. However, there is a need for empirical comparison between adaptive illumination and a fixed illumination baseline to demonstrate its marginal contribution. Second, the depth-AI confidence correlation (section 5.4, *r* = −0.959) suggests that AI confidence is largely a physical property and is a linear approximation for the attenuation in the optical path. Thus, it may be used as an online measurement parameter to decide whether to perform a new pass with re-illumination for very deep holes, as was defined with the correction loop ΔP in Equation 4. Third, the poor correlation between position errors and diameter errors (section 5.4) suggests that it is necessary to keep evaluating, and when required compensating, these errors separately as two different error sources in the systematic error model (Equation 14) rather than assumed to share a single dominant error source. The findings must be viewed in the context of the following boundary conditions. The validation described in section 5 covers a range of hole diameters from 5 to 15 mm, depths of 10–45 mm (depth-to-diameter ratios of 2.0–3.1), three distinct reflectivity categories, and angles of incidence up to 20°; It is difficult to make any extrapolation beyond these parameters, especially as we have no data for depth to diameter ratios higher than 3.1 and for ratios of 5–10. This is the area that the deep-hole optical inspection literature considers critical for occlusion problems, but it is not considered in the present research and is defined as priority future research in section 6. Moreover, CMM measurement technique, robot calibration precision, and diversity of training data for AI are regarded in this research as preconditions that are set in accordance with CMM performance criteria and machine-learning generalization considerations stated in section 2, rather than as variables investigated independently.

## 6 Conclusion

In this paper, a robotic metrology system using adaptive optics in tandem with AI has been proposed for contactless measurement of geometries of blind holes. This system comprises of six functional layers—a six axes robotic arm, a photonic measurement unit, an adaptive light-emitting unit, an AI vision unit, sub-pixel geometrical reconstruction, and robot-aware coordinate transformation and error compensation—into a single measurement loop formalized via a set of governing equations encompassing the diffuse reflection illumination model, illumination quality minimization objective, AI segmentation and the corresponding loss function, sub-pixel circle/ellipse fitting, robot-camera homogeneous transformation, systematic error compensation model, and the uncertainty budget. For 30 trials where holes had diameters between 5 mm to 15 mm, depths of 10–45 mm, three different reflectivities of surfaces, and robot approaches angles from 0° to 20°, the framework was able to achieve a detection rate of 100%, a three-dimensional position error of 0.0322 mm (95% bootstrap CI 0.028–0.037 mm; root mean square error (RMSE) 0.0344 mm, maximum 0.0645 mm), an absolute diameter error of 0.0544 mm (RMSE 0.0647 mm), repeatability standard deviation of 0.0217 mm, and a mean AI confidence of 0.935. With increased reflectivity on the object surface, the adaptive lighting controller lowered the emitted amount of light, which resulted in stabilizing the value of positioning errors within the 0.026–0.037 mm range regardless of the class of reflectivity, On the other hand, hole depth was found to be the major cause of the reduced confidence in AI through the use of correlation analysis (*r* = −0.959). All the observed errors did not exceed the 1 mm submillimeter accuracy level used in this experiment. The above results are based on a proof-of-concept dataset with relatively small sample size (*n* = 30; depth to diameter ratios between 2.0 and 3.1). Error in position was roughly 50% greater when approach angles were ≥15° (mean error 0.041 mm compared to 0.028 mm), there is a confounding effect of reflectivity and approach angle in the experimental design. These preliminary findings suggest that considering light as an active control parameter instead of a passive imaging tool, together with the use of artificial intelligence for segmentation, sub-pixel geometric modeling and robot-based calibration, may be a feasible approach to autonomous and noncontact robotic metrology of geometrically inaccessible blind holes. The architecture suggested herein has direct application in the field of in-process inspection of aerospace structures, automotive engines and hydraulic parts, molds and dies inspection, turbine components, additive manufactured parts, and machined assemblies. Its major strength is the ability to use the same robotic system for both manufacturing and inspection without having to transfer the component to a CMM.

### 6.1 Limitations

However, some of the weaknesses of the proposed system should be mentioned. The first one is that the optical detection is still influenced by the surface reflectivity and scattering. For the ratio of hole depth to its diameter larger than 5–10, optical occlusion effect becomes important and cannot be compensated for by adaptive lighting. Second, the effectiveness of the AI-based segmentation algorithm is strongly dependent on the representativeness of the training dataset in terms of material type, hole depth and diameter, surface reflectivity, lighting, and measurement geometry. Moreover, robot placement inaccuracies might be more pronounced when there is an optical field of view that is narrower, making it necessary to periodically calibrate the extrinsic transformation matrix (ETC). Additional parameters that might impact measurement results in practice during machining operations include surface roughness, burrs, contaminants, oxidation, and deviations from roundness. These have not been considered in this validation study. Dimensional traceability needs to be determined for industrial applications via calibration using artifact measurements performed using the CMM, as shown in Equation 19. The statistical scope of this validation study is constrained by the 30 unreplicated experiments performed using a partially crossed experiment. The ratio of depth-to-diameter under investigation was limited to 2.0–3.1, since there were only nine tests where d/D ≥ 3.0, and only 10 tests where the angle of approach was at least 15°. Moreover, there was a positive correlation between hole depth and hole diameter (*r* = 0.86), as well as reflectivity and angle of approach *r* = 0.74. Thus, the influence of these parameters cannot be considered separately, and correlations presented in section 5.4 have to be viewed not as causal relations, but rather as associations found in the dataset being investigated. The measurements obtained by CMM were used as reference measurements for testing the suggested system.

### 6.2 Future work

The future works will include detailed experiments using hardware to measure the role played by AI-based segmentations, adaptive lighting, sub-pixel fitting, and robot error correction individually. In the future, more experimental data will be used that is well-designed to cover a larger variety of hole depths, ratio of depth-to-diameter, surface reflectivity, approach angles, and materials. Furthermore, the experiments will also include comparison of the proposed technique with other setups including fixed lighting and image processing. Further validation of metallic and industrial parts, particularly those with difficult deep hole geometry, will be necessary for analyzing the generality and reliability of the proposed approach. This work will contribute toward understanding the module performance, minimizing the uncertainty, and the transition of the proposed robotic metrology system toward the automated inspection applications.

## Data Availability

The datasets presented in this study can be found in online repositories. The names of the repository/repositories and accession number(s) can be found at: https://github.com/GirirajMannayee/AI_Robotic_Blind_Hole.
